# Efficacy and safety analysis of massage and medium-frequency electrotherapy in the treatment of congenital muscular torticollis in infants

**DOI:** 10.3389/fped.2025.1594911

**Published:** 2025-10-01

**Authors:** Kai-nan Lin, Ai-jing Sun, Xiang-xuan Wang

**Affiliations:** ^1^ Department of Pediatric Orthopedics, Fujian Children’s Hospital (Fujian Branch of Shanghai Children’s Medical Center), College of Clinical Medicine for Obstetrics & Gynecology and Pediatrics, Fujian Medical University, Fuzhou, China; ^2^ Aofeng Community Health Service Center, Taijiang District, Fuzhou, Fujian, China

**Keywords:** infants, congenital muscular torticollis, massage therapy, medium-frequency electrotherapy, treatment efficacy, safety

## Abstract

**Objective:**

To evaluate the efficacy and safety of massage combined with medium-frequency electrotherapy (M&MFE) in the treatment of congenital muscular torticollis (CMT) in infants.

**Methods:**

We retrospectively analyzed the clinical data of 48 CMT patients who underwent M&MFE treatment at the Fujian Children's Hospital and Traditional Chinese Medicine Department of Ao-feng Community Health Service Center in Tai-jiang District, Fuzhou City, from May 2021 to December 2023. All patients had complete follow-up data, received continuous treatment for 6 months, and were followed up for at least 1 year. The following parameters were evaluated: cervical range of motion (ROM) and muscle function scale to assess efficacy, and complications during treatment were recorded.

**Results:**

A total of 48 patients were included, comprising 26 females and 22 males. The age at initial treatment ranged from 21 to 135 days, with a mean of (41.2 ± 25.4) days. There were 21 cases with left-side involvement and 27 cases with right-side involvement. All patients were followed up for 13–20 months, with a mean follow-up duration of (14.5 ± 2.6) months. By the end of follow-up, 5 patients (10.4%) underwent surgical release. The overall efficacy rate was 89.6%, and significant improvements were observed in cervical ROM and muscle function scores after treatment (*P* *<* *0.05*). No complications related to the skin, blood vessels, nerves, or muscles occurred during the treatment period.

**Conclusion:**

The combination of manual massage and local medium-frequency electrotherapy is a safe and effective non-surgical treatment option for treating infants with congenital muscular torticollis. It significantly improves the neck range of motion and function, and has high clinical promotion value.

## 1 Introduction

Congenital muscular torticollis (CMT) is a common congenital musculoskeletal disorder, with an incidence ranging from 0.3% to 2.0% (1). It is typically caused by fibrosis and pathological contracture of the unilateral sternocleidomastoid muscle (SCM), leading to restricted neck movement, characterized by head tilt to one side and rotation to the opposite side. Severe cases can result in head and facial asymmetry, strabismus, and cranial and spinal deformities (2). If not treated promptly and effectively, the deformity can progressively worsen, significantly affecting the child's appearance and function.

The exact etiology of CMT is still unclear (3), with potential causes including prenatal factors (birth trauma, abnormal intrauterine position), SCM fibrosis (infectious myositis, perinatal hemorrhage), and primary myopathy. Clinical practice guidelines (4, 5) recommend physical therapy as the first-line treatment for CMT. In addition to physical therapy, other commonly used methods include traditional Chinese medicine, manual stretching, local drug injection, corrective braces, therapeutic devices, and home rehabilitation (6). For older children who do not respond to non-surgical treatment, surgical release of the contracted SCM may be necessary (7). Massage is widely used in the treatment of CMT, but there are various theories and no standardized operating method. In recent years, the kneading and stretching technique proposed by Professor Zhang Shi-qing (8) has shown promising results in the treatment of CMT. Studies have indicated (9) that this method can reduce neck masses, improve treatment efficacy and cure rates, and shorten the treatment duration.

Medium-frequency electrotherapy, using 1–100 kHz alternating currents, can be classified into interferential current therapy, constant amplitude medium-frequency electrotherapy, modulated medium-frequency electrotherapy, and low-medium frequency mixed therapy based on waveform and frequency. It promotes local blood circulation, relieves pain, softens scars, and releases adhesions. Medium-frequency electrotherapy has been reported to treat neurological diseases and relieve somatic pain (10).

In recent years, the combination of massage and medium-frequency electrotherapy has gained attention as a potential treatment for CMT. Massage therapy, through manual manipulation, can improve muscle flexibility and blood circulation, while medium-frequency electrotherapy can further enhance these effects by promoting deep tissue relaxation and reducing muscle stiffness. The research conducted by Dong Rak (11) indicates microcurrent therapy may increase the efficacy of therapeutic exercise with ultrasound for the treatment of congenital muscular torticollis involving the entire sternocleidomastoid muscle. Some studies (12) have also pointed out massage, and frequency-specific microcurrent may be effective in congenital muscular torticollis treatment. However, there is limited evidence on the efficacy and safety of this combined approach in infants with CMT.

There is currently no detailed description of the clinical efficacy of massage combined with medium-frequency electrotherapy in the treatment of CMT in infants. This study collected clinical data from patients treated with M&MFE for CMT at our institution from May 2021 to December 2023, aiming to explore the efficacy and safety of massage combined with medium-frequency electrotherapy in the treatment of CMT in infants, providing new evidence for conservative treatment.

## 2 Materials and methods

### 2.1 Inclusion and exclusion criteria

#### 2.1.1 Inclusion criteria

1. Diagnosed with congenital muscular torticollis by a pediatric orthopedic specialist, including mass and non-mass types;
2. Age ≤ 1 year;
3. Regularly underwent M&MFE treatment at our department with complete medical records;
4. Legal guardians agreed to the treatment plan and signed informed consent.

#### 2.1.2 Exclusion criteria

1. Imaging studies indicating other causes of torticollis (congenital cervical disorders, traumatic cervical injury, inflammation, or visual impairment);
2. Presence of ulcers, scars, or skin damage on the neck unsuitable for treatment;
3. Associated with other congenital malformations or severe systemic diseases;
4. Previously received other treatments;
5. Incomplete follow-up data.

### 2.2 General information

A retrospective analysis was conducted on the medical records of CMT patients treated at the Fujian Children's Hospital and Traditional Chinese Medicine Department of Ao-feng Community Health Service Center in Tai-jiang District, Fuzhou City, from May 2021 to December 2023. All infants received SCM stretching combined with M&MFE treatment for 6 months, with at least 1 year of follow-up.

A total of 48 patients met the inclusion and exclusion criteria, including 26 females and 22 males. The age at initial treatment ranged from 21 to 135 days, with a mean of 41.2 ± 25.4 days. There were 21 cases with left-side involvement and 27 cases with right-side involvement. 37 infants were delivered vaginally, and 11 were delivered by cesarean section. 44 infants were full-term, and 4 were preterm. All patients had palpable masses in the SCM. All patients were followed up for 13–20 months, with a mean follow-up duration of (14.5 ± 2.6) months. Relevant demographic characteristics are shown in Table 1.

**Table 1:** Demographic and clinical characteristics of patients with congenital muscular torticollis.

Item		*N* (%)
Age (*x¯* ± *s*, days)		(41.2 ± 25.4)
Gender	Female	26 (54.2%)
	Male	22 (45.8%)
Side	Left	21 (43.2%)
	Right	27 (56.8%)
Type of delivery	Natural birth	37 (77.1%)
	Cesarean section	11 (22.9%)
Full-term birth	Yes	44 (91.7%)
	No	4 (8.3%)
Muscle mass		48 (100%)
Follow-up time (x¯ ± s, months)		(14.5 ± 2.6)
Surgical treatment		5 (10.4%)

This study was conducted in strict accordance with the STROBE statement. The Medical Ethics Committee of our institution provided a waiver of approval for this study, and all guardians of the infants provided informed consent.

### 2.3 Diagnostic criteria

The diagnosis of CMT was based on the “Physical Therapy and Evidence-Based Clinical Practice Guidelines for Congenital Muscular Torticollis” (5, 13) and “Zhu Fu-tang's Practical Pediatrics” (14):

1. Spindle-shaped mass or localized muscle tension and contracture in the affected SCM;
2. Head tilt to the affected side, chin rotation to the healthy side, and varying degrees of restricted neck rotation and tilt to the healthy side;
3. Ultrasound showing thickening or increased/decreased echogenicity of the affected SCM, or detectable muscular mass.

Meeting all three criteria confirms the diagnosis.

### 2.4 Treatment methods

Infants received treatment three times a week at our department. Each session included two parts: stretching and massage, conducted by experienced acupuncturists and massage therapists for 20–30 min. After a 15 min rest, medium-frequency electrotherapy was administered by trained personnel for 5 min.

#### 2.4.1 SCM stretching

According to CMT guidelines (5), painless stretching of the SCM was performed to improve its extensibility. The stretching protocol in this study was as follows: 10 stretches per set, with each stretch lasting 5–10 s and a 10-second rest between sets, repeated for 5 sets.

All infants were stretched in the supine position by two people, with one stabilizing the infant's shoulders and the other standing above the head, stretching within an appropriate range to ensure adequate blood flow to the muscles, with a rotation range <90°.

#### 2.4.2 Massage therapy

Infants were placed flat on the treatment table without a pillow, with shoulders aligned with the edge of the table, head turned to the healthy side, fully exposing the entire SCM. The massage therapist stood at the infant's head.

The therapist used the index and middle fingers to gently knead the affected SCM, its insertion points, and surrounding muscles for 3–5 min.

For infants with masses, the therapist gently pinched the mass for 3–5 min.

The infant was then placed in the prone position, and the therapist gently massaged the trapezius and scapular muscles for about 2 min.

Parents could accompany and comfort the infant during treatment. Infants were advised not to be full before treatment, and the procedure was performed gently to avoid pressure on the throat and neck vessels, with close monitoring of the infant's breathing and vomiting. If vomiting or severe crying occurred, the procedure was immediately paused.

#### 2.4.3 Medium-frequency electrotherapy

Medium-frequency electrotherapy for infants must be performed under the guidance of a professional doctor, considering the special physiological characteristics and specific conditions of infants, and used with caution (zp-100CHII type). The parameter settings for medium-frequency electrotherapy in children require careful adjustment under the guidance of professional rehabilitation therapists, based on age, tolerance, and therapeutic objectives. This study recommends the following baseline parameters, which should be dynamically modified according to individual patient conditions: Carrier frequency: 2,000 Hz; Modulation frequency: 10–50 Hz; Intensity: 5–20 mA; Waveform: Biphasic square-wave pulses; Pulse width: 150 μs; Output mode: 20 s on/1 s off. The treatment steps were as follows:

##### 2.4.3.1 Pre-treatment assessment and preparation

1. Infant assessment: Check the overall condition of the infant and observe for any skin damage or infection.
2. Equipment check: Ensure the medium-frequency electrotherapy device is functioning properly, with no damage or aging of the electrodes, and stable and precise current output.
3. Equipment disinfection: Clean and disinfect the electrodes and other parts that come into contact with the infant's skin to prevent cross-infection.

##### 2.4.3.2 Treatment process monitoring

1. Parameter adjustment: Adjust the current intensity and treatment time based on the infant's age, weight, and physical condition. The treatment time was gradually increased based on the infant's tolerance, with treatment time for infants under 3–6 months controlled at around 5 min.
2. Close observation: Monitor the infant's response, such as crying, restlessness, skin redness, or rash. If these symptoms occurred, treatment was immediately stopped, and appropriate measures were taken.
3. Standardized operation: The electrodes were placed on the SCM mass, ensuring close contact with the skin and avoiding gaps. Care was taken to avoid the carotid sinus. During treatment, the infant's movement was minimized to prevent electrode displacement.

##### 2.4.3.3 Post-treatment care

1. Infant assessment: After treatment, the electrodes were removed, and the infant's skin was checked for abnormalities. Mild redness was observed, while swelling or burns were treated with cold compresses and recorded in detail.
2. Skin cleaning: The treatment area was cleaned with warm water to remove any residual gel from the electrodes, and an appropriate amount of baby-specific lotion was applied to protect the skin.
3. Observation of reactions: The infant's overall response, including mental state and limb activity, was observed. If lethargy or abnormal limb activity occurred, it was promptly addressed and recorded.

### 2.5 Follow-up and clinical efficacy evaluation

During the follow-up stage, two staff members conducted standardized monitoring for the included patients. They followed up at the predetermined time points using detailed case report forms.

#### 2.5.1 Follow-up

Cervical ROM (including lateral flexion and rotation) and muscle function scale were used to evaluate the clinical efficacy of the treatment (20). Follow-up was conducted for at least 1 year from the initial treatment. Surgical evaluation: If neck passive movement was restricted with rotation >6° or lateral flexion >6° after treatment, surgical indications were assessed by a specialist.

#### 2.5.2 Cervical ROM assessment

A specialized goniometer was used to measure the head tilt angle and ROM (lateral flexion and rotation) in the supine position. Head tilt was defined as the angle between the head midline and body midline (15).

#### 2.5.3 Functional recovery assessment

The muscle function scale (16, 17) was used to assess bilateral muscle function. The score ranged from 0 to 5, and for statistical analysis, scores were recorded from 1 to 6. The difference of Muscle Function Scale Score = Affected side score–unaffected side score.

#### 2.5.4 Complications during treatment

Complications related to the skin, blood vessels, nerves, or muscles during M&MFE treatment were recorded.

### 2.6 Statistical analysis

SPSS 22.0 (IBM, USA) was used for statistical analysis. Normally distributed measurement data were expressed as mean ± standard deviation (*x¯* ± *s*); count data were expressed as numbers and percentages (%), and comparisons were made using the chi-square test; ranked data comparisons were made using the rank-sum test. *P* < 0.05 was considered statistically significant.

## 3 Results

All 48 patients was followed up for 13–20 months, with a mean follow-up duration of (14.5 ± 2.6) months. Among the 48 infants, 5 (10.4%) underwent surgical release due to poor treatment response, while the remaining 43 (89.6%) showed significant improvement with conservative treatment.

**Figure 1:**
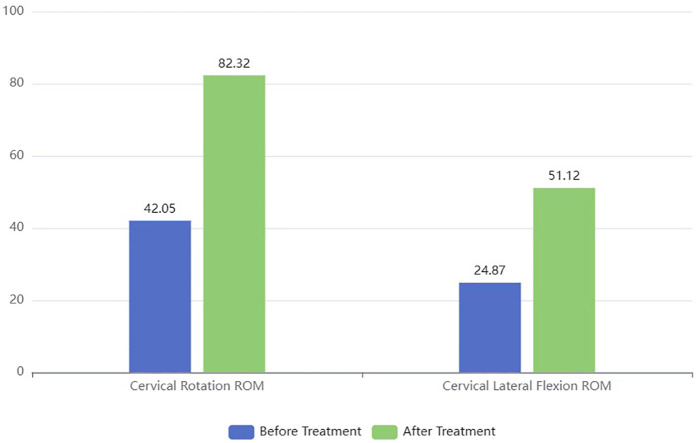
Changes in the cervical range of motion.

Cervical ROM: After M&MFE treatment, the infants' cervical ROM (including lateral flexion and rotation) significantly improved (*P* = 0.03, Table 2). The significant improvement in cervical ROM (both rotation and lateral flexion) after treatment indicates that the combined therapy effectively restored neck mobility, which is crucial for the normal development of infants with CMT (Figure 1).

**Table 2:** Comparison of cervical ROM and muscle function scale scores before and after treatment.

Item	Before treatment	After treatment	*P*-value
Cervical Rotation ROM (°)	42.05 ± 2.27	82.32 ± 9.43	0.03
Cervical Lateral Flexion ROM (°)	24.87 ± 6.43	51.12 ± 2.53	0.02
The difference of MFS^a^	2.35 ± 0.68	0.18 ± 0.75	0.02

ROM, range of motion; MFS, Muscle Function Scale Score.

^a^ The difference of MFS = Affected side score–unaffected side score.

The difference of Muscle Function Scale Score: After M&MFE treatment, the muscle function scale score significantly improved (*P* = 0.02, Table 2).

Complications: All patients experienced mild skin redness after M&MFE treatment, which resolved within 10 min after the procedure. No severe complications related to the skin, blood vessels, nerves, or SCM muscles occurred.

## 4 Discussion

CMT is a common musculoskeletal disorder in children, primarily caused by unilateral SCM contracture, leading to head tilt to the affected side and facial rotation to the healthy side. If not treated promptly, it can not only affect the child's appearance but also lead to facial asymmetry, cervical scoliosis, and other secondary deformities as the child grows, significantly impacting their physical and mental health and quality of life (2). Early diagnosis and appropriate treatment can yield good results, with earlier treatment leading to better outcomes. In this study, most infants started treatment before 3 months of age, and the overall effective rate of M&MFE treatment was 89.6%. Among the 5 patients who underwent surgical release, 3 were older than 6 months at the time of initial treatment, suggesting that early intervention may be crucial for the success of conservative therapy. Further studies are needed to identify the factors associated with treatment failure in older infants.

Previous studies have confirmed (15) that physical therapy can reduce the physical stiffness of fibrotic masses in CMT infants and effectively improve cervical ROM. SCM stretching can increase muscle length and elasticity and is an effective conservative treatment for CMT in children (16). Massage (18, 19), as a traditional treatment method, is widely used. Professional techniques such as kneading, pinching, and stretching the contracted SCM can promote local blood circulation, release muscle adhesions, correct muscle fiber alignment, and improve contracture. Medium-frequency electrotherapy promotes local blood circulation, relieves pain, softens scars, and releases adhesions, and has been used to treat neurological diseases and relieve somatic pain (10).

The mild skin redness observed in all patients was transient and resolved within 10 min, indicating that the treatment is well-tolerated by infants. However, future studies should monitor for potential long-term skin effects, especially in infants with sensitive skin.

This study combined massage with medium-frequency electrotherapy in the treatment of CMT in infants and achieved promising results. Medium-frequency electrotherapy uses medium-frequency currents to penetrate deep into tissues, generating a thermal effect that further promotes local blood circulation, accelerates metabolism, and stimulates muscle contraction, enhancing muscle strength. It synergizes with massage to release adhesions and relieve contractures. The study results showed that after combined treatment, the degree of head tilt, SCM contracture, and neck movement restriction significantly improved, demonstrating the complementary effects of the two treatment methods in promoting recovery. More prospective studies with a big sample size are needed to buttress this finding.

In children with CMT who do not receive effective treatment in the early stages, craniofacial deformities may gradually develop. The primary manifestations include facial asymmetry, with unequal distances from the lateral canthus to the oral commissure bilaterally—the affected side exhibits a shortened distance, while the unaffected side shows elongation. The ocular plane on the affected side is lower, and due to the misalignment of the two eyes, visual fatigue and subsequent visual impairment may occur. The unaffected side of the face appears rounded and full, whereas the affected side is narrow and flat. Additionally, asymmetric changes may involve the entire face, including the nose and ears. Although this study did not specifically assess facial asymmetry in patients, we observed that none of the children treated with massage combined with medium-frequency electrotherapy developed craniofacial deformities by the end of follow-up. However, further investigation with extended follow-up periods is required to confirm the impact of this treatment regimen on facial symmetry in affected children.

Despite the promising results, this study has several limitations. First, this study was limited by its single-center retrospective study designed with a small sample size. The enrolled cohort of congenital muscular torticollis (CMT) patients did not comprehensively represent the full spectrum of disease severity or age groups, which may introduce limitations to the findings. To address this, our research team plans to conduct multicenter, large-scale studies with expanded sample sizes to further validate the long-term efficacy and safety of massage combined with medium-frequency electrotherapy, thereby generating more robust evidence to support our current conclusions. Second, only short-term clinical outcomes were evaluated in this study, while long-term prognosis remains undetermined. Enrolled patients will undergo scheduled follow-up assessments to evaluate the sustained therapeutic effects of the intervention. When feasible, subsequent studies will report long-term follow-up data and comprehensively assess the treatment's impact on infant growth and development. Third, given the retrospective nature of this study, inherent limitations such as potential selection bias and confounding factors during patient enrollment must be acknowledged. However, all included patients were definitively diagnosed by specialist physicians and received standardized treatment from the same acupuncture and tuina therapist, substantially mitigating selection bias. Finally, the absence of a control group may constrain comparative efficacy analyses between tuina combined with medium-frequency electrotherapy and alternative treatment plans. Our team fully recognizes these limitations. Nevertheless, analysis of the enrolled patients demonstrated exceptional clinical efficacy of this combined therapy for congenital muscular torticollis. Given these encouraging findings, we deemed it imperative to promptly disseminate these results for peer discussion.

In conclusion, massage combined with medium-frequency electrotherapy is a safe and effective treatment for CMT in infants, demonstrating significant advantages and providing new ideas and methods for clinical treatment. However, given the limitations of this study, further in-depth research is needed to continuously optimize the treatment plan, bringing benefits to more infants.

## Data Availability

The raw data supporting the conclusions of this article will be made available by the authors, without undue reservation.
